# Detection of CRISPR-Cas9-Mediated Mutations Using a Carbon Nanotube-Modified Electrochemical Genosensor

**DOI:** 10.3390/bios11010017

**Published:** 2021-01-08

**Authors:** Ezgi Kivrak, Tekle Pauzaite, Nikki A. Copeland, John G. Hardy, Pinar Kara, Melike Firlak, Atike I. Yardimci, Selahattin Yilmaz, Fahreddin Palaz, Mehmet Ozsoz

**Affiliations:** 1 Department of Analytical Chemistry, Faculty of Pharmacy, Ege University, Izmir 35100, Turkey; 91180001152@ogrenci.ege.edu.tr; 2 Department of Biomedical and Life Sciences, Faculty of Health and Medicine, Lancaster University, Lancaster LA1 4YQ, UK; tp471@cam.ac.uk (T.P.); n.copeland@lancaster.ac.uk (N.A.C.); 3 Department of Chemistry, Faculty of Science and Technology, Lancaster University, Lancaster LA1 4YB, UK; j.g.hardy@lancaster.ac.uk (J.G.H.); mfirlak@gtu.edu.tr (M.F.); 4 Materials Science Institute, Lancaster University, Lancaster LA1 4YB, UK; 5 Department of Chemistry, Gebze Technical University, Gebze 41400, Turkey; 6 Department of Chemical Engineering, Izmir Institute of Technology, İzmir 35430, Turkey; atike.yardimci@usak.edu.tr (A.I.Y.); selahattinyilmaz@iyte.edu.tr (S.Y.); 7 Faculty of Medicine, Hacettepe University, Ankara 06100, Turkey; fahreddinpalaz@hacettepe.edu.tr; 8 Faculty of Engineering, Near East University, Lefkoşa 99138, Turkey

**Keywords:** CRISPR-Cas9, homology-directed repair (HDR), electrochemical genosensor, mutation detection, carbon nanotube-modified PGE

## Abstract

The CRISPR-Cas9 system has facilitated the genetic modification of various model organisms and cell lines. The outcomes of any CRISPR-Cas9 assay should be investigated to ensure/improve the precision of genome engineering. In this study, carbon nanotube-modified disposable pencil graphite electrodes (CNT/PGEs) were used to develop a label-free electrochemical nanogenosensor for the detection of point mutations generated in the genome by using the CRISPR-Cas9 system. Carbodiimide chemistry was used to immobilize the 5′-aminohexyl-linked inosine-substituted probe on the surface of the sensor. After hybridization between the target sequence and probe at the sensor surface, guanine oxidation signals were monitored using differential pulse voltammetry (DPV). Optimization of the sensitivity of the nanogenoassay resulted in a lower detection limit of 213.7 nM. The nanogenosensor was highly specific for the detection of the precisely edited DNA sequence. This method allows for a rapid and easy investigation of the products of CRISPR-based gene editing and can be further developed to an array system for multiplex detection of different-gene editing outcomes.

## 1. Introduction

The analytical performance of biosensors has been greatly enhanced through the development of nanomaterials by nanotechnology engineering [1]. Due to the small size of nanomaterials, great successes have been achieved in the microfabrication of electrochemical biosensors [2,3]. Carbon nanotube-modified disposable pencil graphite electrodes (CNTs) are highly effective in signal amplification because of their excellent conductivity and are frequently used in biosensors as immobilization of biomolecules on CNTs leads to increases in the signal [4]. The incorporation of nanomaterials and bioanalytical chemistry also led to the emergence of genosensing devices [5]. Electrochemical transduction in genosensing facilitates sequence-specific interrogation of target DNA sequences. Electrochemical genosensing methods require minimal instrumentation and enable rapid and straightforward analysis of DNA sequences with high selectivity and sensitivity [6,7,8].

The emergence of clustered regularly interspaced short palindromic repeats (CRISPR)–CRISPR-associated protein 9 (Cas9) genome engineering technology has facilitated editing the genome of any species [9]. Cas9 endonuclease cuts the target DNA with the help of a trans-activating CRISPR RNA (tracrRNA) and a CRISPR RNA (crRNA), which provides sequence specificity through the formation of Watson and Crick base pairs with the target DNA site [10]. Recognition of the target DNA sequence next to the protospacer adjacent motif (PAM) allows Cas9 to activate and cut the target DNA [11]. In mammalian cells, Cas9-induced double-strand breaks are mostly repaired using either nonhomologous end-joining (NHEJ) or homology-directed repair (HDR) pathways that can be utilized to delete or mutate target genes. NHEJ-mediated repair is error-prone often leads to insertions or deletions (indels), which is desired for gene knockout. The more precise HDR pathway uses template DNA to repair damaged DNA, which can be utilized to introduce precise edits into genomes [12]. CRISPR-Cas systems can also be used for the detection of nucleic acids, proteins, and small molecules [13,14,15,16,17,18]. By using these biosensors, it is possible to develop new point-of-care diagnostics for pathogen detection, genotyping, cancer mutation detection, and disease diagnosis [19].

In addition to CRISPR-dependent HDR, there are some other CRISPR-Cas-based tools that are precise in gene editing and do not depend on double-strand break repair mechanisms. For example, CRISPR base editors can introduce single base substitutions to target DNA sites in a programmable manner without double-strand break formation [20,21]. In addition, recently developed Prime Editors can be used to create targeted point mutations, insertions, deletions, and combinations of these edits at target DNA sites [22]. Although methods such as HDR-based genome editing, base editing, and prime editing are precise, they are not fully efficient and may introduce undesired edits into the target DNA. Therefore, it is important to carefully examine the outcomes of any CRISPR assay for safer and more efficient genome editing applications [23,24]. CRISPR-based genome editing outcomes can be analyzed using PCR amplification of target site followed by DNA sequencing, or restriction fragment length polymorphism (RFLP) analysis can be conducted after PCR [25].

Inosine-substituted probes have been used for the detection of genomic mutations [5,26]. The hybridization between the probe and denatured PCR amplicons can be determined by measuring the oxidation signal of guanine in connection with differential pulse voltammetry (DPV). This method enables the detection of DNA sequences with high sensitivity and single-base specificity [26,27]. It has been shown that inosine is less electrochemically reactive than the guanine base. Thus, effective hybridization sensors could be realized by immobilization of probe strands in which inosine was substituted for guanine. High catalytic currents are provided only after hybridization to guanine-containing target strands [28]. In the light of this phenomenon, yes/no genosensing platforms have been developed for the detection of mutations due to the guanine signal appearance after the hybridization [27,29].

Here for the first time, electrochemical nanogenosensing based on inosine-substituted probes and disposable pencil graphite electrodes was used to verify the presence of CRISPR-Cas9-introduced mutations in murine cells. This method allows for efficient detection of the desired outcome created by HDR in CRISPR-Cas9 studies with high selectivity and specificity.

## 2. Materials and Methods

### 2.1. Apparatus

The differential pulse voltammetry (DPV) technique was used for analysis with an Autolab PGSTAT-30 (Eco Chemie, Utrecht, The Netherlands) electrochemical analysis system and GPES 4.9 software package (Eco Chemie, The Netherlands). A three-electrode system involving disposable graphite electrodes (PGEs) with diameters of 0.5 mm and lengths of 3 cm long as working electrodes (Tombo, Osaka, Japan), a platinum wire as the counter electrode and a reference electrode (Ag/AgCl).

### 2.2. Reagents and Solutions

*N*-hydroxysulfosuccinimide sodium salt (NHS), [*N*-(3-dimethylamino)propyl)]–N′-ethylcarbodiimide (EDC) and Trizma hydrochloride were purchased from Sigma-Aldrich Chemical Company (Taufkirchen, Germany), Sodium dodecyl sulfate (SDS) and tri-sodium citrate were purchased from Merck (Darmstadt, Germany). Other chemicals were of analytical reagent grade and supplied by Merck and Sigma. All experiments were performed at 25 °C. The synthetic oligonucleotides were provided from Invitrogen (Carlsbad, CA, USA) by Thermo Fisher Scientific as a lyophilized powder. The base sequences of the oligonucleotides are as follows (I = inosine):

- S-331 genome wild-type (WT) probe: 5′-NH_2_-C_6_-CICtAAITICTCTIIAIaIIT-3′
- Synthetic WT target: 5′-ACC**T**CTCCAGAGCACTT**A**GCG-3′
- S-331 genome mutant-type (MT) probe: 5′-NH_2_-C_6_-CICCAAITICTCTIIAICIIT-3′
- Synthetic MT target: 5′-ACC**G**CTCCAGAGCACTT**G**GCG-3′
- Synthetic non-complementary sequence: 5′-GGCAGCGGTGACTATGGCACC-3′
- CRISPR-Cas9-edited S-331 gene PCR amplicon (underlined bases refer to the introduced point mutation region via CRISPR/Cas9 system): 5′-TTAGGGCGATTGGGCCCTCTAGATGCATGCTCGAGCGGCCGCCAGTGTGATGGATATCTGCAGAATTCGCCCTTGGAACTGGGTCAAAGGCCTCTGGGAAGATAGAGCTTTGGTCTTCTTGGATTTGCTGGTTTGTTTTCATTTTTGAGACAATCTTGGCTGACCTAGAACTCACTATGTAGACCAGGCTGGCCTCAACTCTTCAGAAGAGATCCGCCTGTCTCTTCCTCCCTAGGGTCAGGATCAAAGGCATAGACCACCACAACTGGCTTTTTGCTTATCTTTGGATCTTTGCTAGCTCAGAGGAGTCCACCGAGAAAGGCCCTACAGGGCAGCCACAAGCAAGGGTCCAGCCTCAGACCCAGATGACAGCACCAAAGCAGACACAGACCCCGGATCGGCTGCCTGAGCCACCAGAAGTCCAAATGCTGCCGCGTATCCAGCCACAGGCACTGCAGATCCAGACCCAGCCAAAGCTGCTTTGGCTGGGTCTGAGGCAGGCACAGACACAGACCGCTCCAGAGCACTTGGCGCCCCAGCAGGATGTCCTGGAG-3′
- Non complementary PCR amplicon (*E. coli*): 5′-AAAAGTGAAAGCGAACCGAATCTGTTAAATCAGCGAGTTGAGATCAAAAAATCTGACCTTGTTAACTATAATCCGATTGCGGAAAAGCACGTCAATGGGACGATGTCACTGGCTGAGCTTAGCGCGGCCGCGCTACAGTACAGCGATAACGTGGCGATGAATAAGCTGATTGCTCACGTTGGCGGCCCGGCTAGCGTCACCGCGTTCGCCCGACAGCTGGGAGACGAAACGTTCCGTCTCGACCGTACCGAGCCGACGTTAAACACCGCCATTCCGGGCGATCCGCGTGATACCACTTCACCTCGGGCAATGGCGCAAACTCTGCGGAATCTGACGCTGGGTAAAGCATTGGGCGACAGCCAACGGGCGCAGCTGGTGACATGGATGAAAGGCAATACCACCGGTGCAGCGAGCATTCAGGCTGGACTGCCTGCTTCCTGGGTTGTGGGGGATAAAACCGGCAGCGGTGACTATGGCACCACCAACGATATCGCGGTGATCTGGCCAAAAGATCGTGCGCCGCTGATTCTGGTCAC-3′

All oligonucleotide and primer stock solutions (1000 µg/mL) were prepared with ultrapure water (18 MΩ, Millipore, Burlington, MA, USA) and stored at −20 °C until use. Immobilization of probes onto the surfaces of electrodes was achieved via carbodiimide chemistry (5 mM EDC and 8 mM NHS). Dilutions of the capture probe (CP) were prepared with an aqueous acetic acid (0.5 M) with NaCl (20 mM) (ABS; pH = 4.8) buffer. Synthetic target sequences and the PCR products and non-complementary amplicons were diluted with hybridization buffer (HB) containing 5 × saline sodium citrate (SSC) + 0.05% solution (0.75 M NaCl, 75 mM sodium citrate dihydrate, 0.05% SDS), and 1× SSC + 0.1% SDS solution (0.15 M NaCl, 15 mM sodium citrate dihydrate, 0.1% SDS) was used as a washing buffer unless otherwise indicated.

### 2.3. Methods

#### 2.3.1. CRISPR-Cas9-Mediated Site-Directed Mutagenesis of the *CIZ1* Gene

The following oligonucleotides were used for the construction of the guide sequence (S331 5′-AGACCCAGCCAAAGCTGCTGgtttt-3′, 5′-CAGCAGCTTTGGCTGGGTCTcggtg-3′. The sequence in the lower case is complementary to the overhang sequence for insertion, the sequence in the upper case is the crRNA sequence that is complementary to the murine *CIZ1* gene and proximal to a protospacer adjacent motif (PAM). The guide sequence was introduced into the linearized GeneArt^®^ CRISPR nuclease vector-containing Cas9 and the CD4 reporter using the manufacturer’s instructions (Invitrogen). Amplification of the plasmid was performed by transformation of One Shot^®^ TOP10 chemically competent *E. coli*, antibiotic selection and overnight cultures. DNA sequencing of the plasmid was performed to verify the construct (Eurofins). Single strand oligonucleotide-directed mutagenesis was used to introduce mutations into the *CIZ1* gene using HDR with a 98 nt single-stranded oligodeoxynucleotide (ssODN): 5′-GCAGATCCAGACCCAGCCAAGCTGCTGAGGCAGGCACAGACACAGACCgCTCCAGAGCACTTgGCGCCCCAGCAGGATCAGGTAGAGCCACAGGTAC-3′.

**Figure 1:**
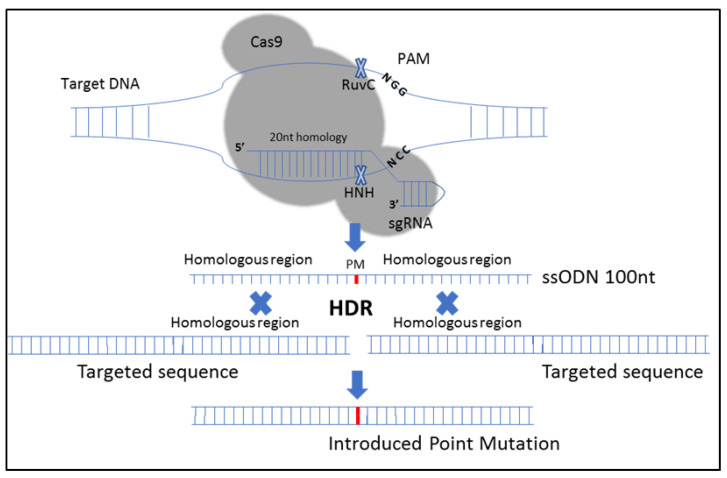
Clustered regularly interspaced short palindromic repeats (CRISPR) RNA (crRNA) was designed with complementary nucleotides adjacent to the protospacer adjacent motif (PAM) to target CRISPR-associated protein 9 (Cas9) endonuclease to the correct DNA sequence within genomic DNA. HNH (an endonuclease domain named for characteristic histidine and asparagine residues) and RuvC-like nuclease domains cut the target DNA 3 nucleotides upstream of the PAM (NGG) sequence leading to the formation of a double-strand break. Mutations were inserted utilizing a homology-directed repair (HDR) pathway to repair the dsDNA breaks using 98 nt single-strand oligodeoxynucleotide (ODN) sequence as a template with 40–50 nucleotides flanking the dsDNA break site to introduce point mutations (PM).

The sequences that contained point mutations (given in lower case) were complementary to the region surrounding the PAM site to aid in efficient HDR. Figure 1 depicts the procedure followed.

Transfection of 3T3 embryonic mouse fibroblast cells with 1 µg of CRISPR-Cas9 expression vector and 2.5 µL of 50 µM ssODN, using 100 µL of transfection reagent Kit-R (Lonza, Basel, Switzerland), was carried out by electroporation using the Nucleofector™ program U-030 (Lonza). Cell enrichment was carried out to isolate the transfected cells with the GeneArt^®^ CRISPR nuclease (CD4 reporter) Vector using Dynabeads^®^ CD4 magnetic beads (Invitrogen). Single cells were plated into a 96-well plate, and mutated cells were identified using a restriction endonuclease specific for desired mutations.

Single cells were cultured in a flat bottomed 96-well plate for 2 weeks, replacing media every 2–3 days and passaging cells on to 24 well plates. Genomic DNA (gDNA) was extracted using 50 μL of QuickExtract™ DNA extraction solution (Epicentre-Lucigen). The sequences of interest were screened after digestion with BanI (S331) restriction enzymes. gDNA was PCR amplified using One Taq Quick-Load 2× master mix (New England BioLabs, Ipswich, MA, USA), 94 °C-30 s, 30 cycles of 94 °C-30 s, 68 °C-30 s, 68 °C-60 s, the final extension was carried out at of 68 °C-5 min. PCR products were DNA sequenced to verify the presence of mutations.

Identification of colonies of interest was identified by restriction endonuclease digestion with BanI according to manufacturer’s instructions. Clones that showed the correct restriction digest pattern were inserted into the TOPO10 Blunt cloning vector (Zero Blunt^®^ TOPO^®^ PCR cloning kit—Thermo Fisher Scientific, Waltham, MA, USA) and DNA sequenced (Eurofins, Luxembourg) for multiple clones to confirm the presence of desired mutations.

#### 2.3.2. Quantitative Determination of Samples by Spectrophotometric Assay

The concentration of all oligonucleotides and PCR amplicons were determined using a spectrophotometric method [30] using a UV-visible Nano Vette microliter cell spectrophotometer (Beckman Coulter, Brea, CA, USA) containing 1 µL sample volume and 0.2 mm path length. 1 A260 unit of double-stranded DNA = 50 µg/mL, and 1 A260 unit of single-stranded DNA = 33 µg/mL.

#### 2.3.3. Synthesis of CNTs

In this study, the carbon nanotubes (CNTs) were synthesized by thermal chemical vapor deposition (CVD) [31] utilizing Co-Mo/MgO nanocatalyst particles fabricated by a gel-combustion strategy [32]. For CNT preparation, the catalyst was put into a quartz vessel that was placed in a quartz container of 1-inch diameter situated in a tubular furnace. The catalyst was reduced under H_2_ flow by incrementing the temperature to 850 °C with a ramp rate of 5 °C/min and stabilized at this temperature for 1 h under 200 standard cubic centimeters per minute (sccm) H_2_. Then, the temperature was set to 1000 °C with the same ramp rate. After this, CNT synthesis was commenced by passing CH_4_ with a flow rate of 50 sccm and H_2_ with 200 sccm over the catalyst. CNT synthesis lasted 40 min under atmospheric pressure. To end the synthesis, the CH_4_ flow was turned off, and the CNTs obtained were left to cool under an atmosphere of H_2_.

#### 2.3.4. Electrode Modification and Probe Immobilization

Prior to probe immobilization, PGE electrodes were activated at 1.40 V for 30 s in acetate buffer solution (ACB, 0.5 M acetic acid, pH 4.8). Probe immobilization was carried out on the surface of the PGEs. The probe and CNTs were allowed to interact in solution as previously described [33]. Briefly, CNTs were added to ACB at a concentration of 1 mg/mL and sonicated for 4 h under ambient conditions. Then, probe DNA was added to the CNT suspension to obtain 5 µg/mL final concentration and the suspension was mixed at 600 rpm for a period of 30 min at 30 °C. Subsequently, each PGE was incubated in separate vials containing 30 µL of the probe-wrapped CNTs for immobilization on the electrode surface over the period of 1 h.

#### 2.3.5. Hybridization and Washing

Denaturation of the PCR amplicons was carried out at 95 °C/8 min and 0 °C/2 min to separate the strands. Hybridization buffers containing the 5 µg/mL of complementary and non-complementary target sequences and amplicons were used for the hybridization between the probe and target sequences. Hybridizations were carried out on electrode surfaces at room temperature by placing the electrodes inside the vials containing 30 µL of the target or non-complementary solutions in a hybridization buffer for 30 min. The electrodes were then immersed in a washing buffer for 1 min to remove the unhybridized DNA.

#### 2.3.6. Voltammetric Transduction

**Figure 2:**
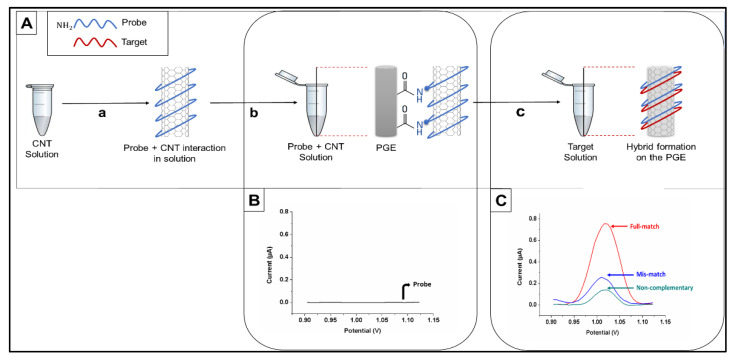
(**A**) Schematic illustration of the electrochemical biosensing of the CRISPR-Cas 9-mediated mutations in the *CIZ1* gene depicting (**a**) CNT suspension and probe interaction in solution phase, (**b**) immobilization of probe-wrapped CNTs onto disposable graphite electrode (PGE) surface, and (**c**) hybridization of probe and target sequences on CNT-modified PGEs. DPV voltammograms obtained after (**B**) inosine-substituted probe immobilization and (**C**) probe and target interactions for the construction of electrochemical “yes/no” platform.

DPV was used to measure the oxidation signal of guanine after placing the electrode in ACB (pH 4.8) by scanning from +0.75 V to +1.40 V at ambient conditions (25 °C). The raw data were obtained as the analytical signal after moving average baseline fitting using a peak width of 0.01 V. All results presented in this paper are the means of at least five measurements, and the error bars show the standard deviations. The procedure for electrochemical biosensing is presented in Figure 2.

## 3. Results

The use of ssODN is highly efficient for introducing small mutations, including single point mutations, via HDR-based gene editing [25,34]. The CRISPR-Cas9 system was used in combination with a 98 nt single-stranded oligodeoxynucleotide (S331ODN) to mutate the single CDK site (S331) within the *CIZ1* protein sequence [35]. *CIZ1* is a protein that cooperates with cyclin A-CDK2 to promote the initiation of DNA replication [36]. A silent mutation was introduced in that region to create a BanI restriction site to screen edited clones. As a result, a total of two point mutations were created in the *CIZ1* sequence by using HDR, including a point mutation that provides the S331A substitution in the *CIZ1* protein and a silent point mutation that allows the formation of a BanI restriction site (Figure 1).

For detection of the wild type and edited DNA sequences, inosine-modified probe sequences complementary to both constructs were designed. Each probe was immobilized onto CNT-modified PGE surfaces covalently, and hybridization reactions were performed with denatured PCR products from WT and mutated *CIZ1* clones (Figure 2A). Amplified oxidation signals of the guanine indicate the formation of a hybrid; therefore, the hybridization was detected by measuring guanine oxidation signals using DPV (Figure 2B,C). *E. coli* PCR amplicons were introduced as negative controls (non-complementary) to determine the selectivity of the designed genoassay. CNT-free ssDNAs were immobilized on the surface of unmodified PGEs, and guanine oxidation signals were found to be 220 nA. However, after the modification of ssDNA with CNTs, the guanine signal increased to 750 nA, resulting in a (3 times) increase over the unmodified PGEs (Figure S2).

**Figure 3:**
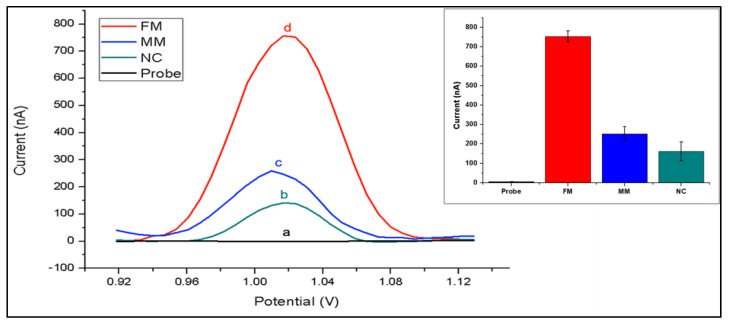
Differential pulse voltammograms of different modified electrodes in 0.5 M ACB: (a) inosine-substituted probe-modified PGE before hybridization; after hybridization with (b) non-complementary sequence (NC), (c) sequence with two mismatched bases (MM) and (d) fully complementary target sequence (FM). Inset: histogram chart of guanine oxidation signals obtained under optimized conditions.

The nanogenosensor was tested using synthetic DNA sequences before using bona fide PCR products. The signal obtained from the synthetic mutant target was substantially higher than the signals obtained from both the synthetic non-complementary sequence and the synthetic wild-type target bearing two mismatches with the mutant probe (Figure 3). These data showed that the mutant probe was highly selective and specific for the detection of the synthetic mutant target.

**Figure 4:**
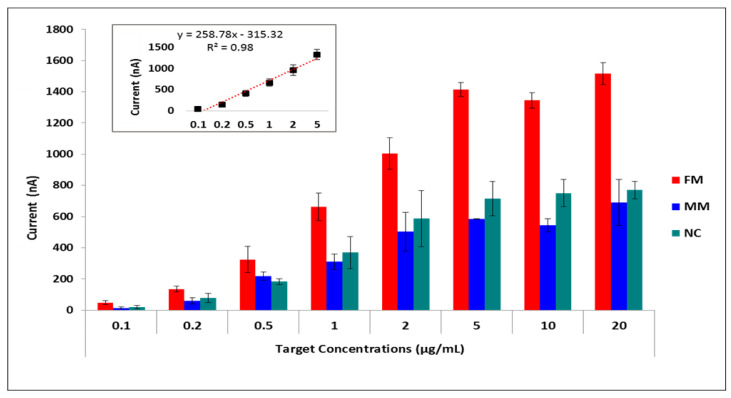
Histograms and linear correlation between the target sequence concentrations ranging from 0.1 to 20 µg/mL at a constant probe concentration of 5 µg/mL on modified genoassay surfaces.

The testing conditions were optimized using synthetic target sequences in order to increase both the selectivity and specificity of hybridization. For selectivity, differences between guanine oxidation signals after hybridization with a complementary target and after hybridization with a non-complementary (FM = full match/NC = non-complementary) synthetic target sequence was obtained. To assess the efficiency of mutation detection, differences in guanine signals obtained from hybridization with a complementary synthetic target and from hybridization with a synthetic target containing two base mutations in the sequence region of interest (FM/MM = mismatch) was obtained. By varying only the probe concentrations ranging from 0.1 to 10 µg/mL, we found that the highest FM/NC and FM/MM values were obtained at 5 µg/mL probe concentration (Table S1). Then we fixed the probe concentration at 5 µg/mL and applied increasing target concentrations ranging from 0 to 50 µg/mL. Hybridization signals increased until the target concentration reached 5 µg/mL, after which the hybridization signal remained almost constant (Figure 4, Table S2). The relative standard deviations of the measurements showed that results were more reproducible and accurate with the lowest error bars at 5 µg/mL target concentration regarding the full-matched hybridization (Mean of the hybridization signal was 1.40 µA with a relative standard deviation of % 1.57 for 5 µg/mL target concentration). Furthermore, the effects of hybridization buffer, washing buffer, and hybridization time were studied (Figure S1 and Tables S3–S5). A hybridization buffer containing 5× SSC +% 0.05 SDS, washing buffer containing 1× SSC +% 0.1 SDS, and 1 min washing time was chosen as optimum conditions by means of highest specificity and selectivity (Tables S1–S5).

**Figure 5:**
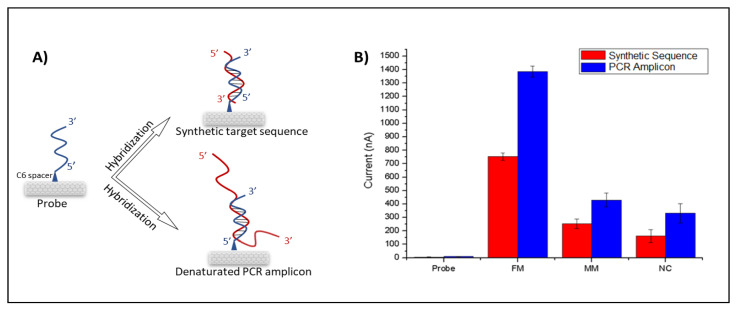
(**A**) Schematic illustration of probe immobilization onto PGE and its hybridization with denatured bona fide PCR amplicon and complementary synthetic target sequence. (**B**) Histogram of voltammetric guanine oxidations signals at +1.0 V obtained for synthetic sequences and denatured PCR amplicons at the same conditions: the probe (before hybridization), FM (probe + target), MM (probe + mismatch target) and NC (probe + non-complementary target).

Finally, the optimized nanogenosensor has been used on real PCR amplicons (Figure 5). The guanine signals of the hybrid tend to further increase when hybridization of probe occurs with PCR amplicons in comparison with the hybridization of the probe with the synthetic target (Figure 5B). This is because the 21-base synthetic target sequence is fully complementary to the 21-base probe sequence. However, hybridization between the probe and the PCR amplicon occurs only between the region that contains the 21-base of the complementary sequence to the probe among the 554-base of the PCR amplicon (Figure 5A). The residual guanines of PCR amplicons not taking part in the hybridization resulted in the increment of the guanine oxidation signals (Figure 5B). Notice that the probe sequence has no oxidation signal by itself because it contains inosine bases instead of guanines. The lowest signal after hybridization was obtained following the probe and non-complementary strand due to their lack of complementarity. The probe and fully matched target have the highest signal after hybridization due to their complementarity. However, a decrease in the hybridization signal was observed between the probe and the mismatched target. The mean and the RSD values of each parameter are given in Table S6. These results show that the nanogenosensor developed here is highly selective and specific for the desired CRISPR-Cas9-based precise gene editing outcome.

## 4. Discussion

Electrochemical biosensing methods at the DNA level are used in a broad range of areas from disease-causing organisms to mutated genes and even detection of food contaminants, DNA/drug interactions and monitoring of the environment. The principle of the proposed detection strategy relies on a well-established method based on electrochemical genosensors. On the other hand, the emergence of the CRISPR/Cas9 system paved the way to alter the genome and control gene expression. The outcomes of any CRISPR-Cas9 assay should be investigated to perform safe and effective genome editing. This study demonstrates the possible use of inosine-substituted probes along with CNT/PGEs for label-free electrochemical genosensing of CRISPR-Cas9-mediated genomic mutations. The techniques used in this study are based on established genosensing methods capable of detecting nucleic acids with high sensitivity and sequence-specificity [5,8,26,33,37]. In this method, a high level of sensitivity and specificity results from performing PCR and hybridization of PCR products with the sequence-specific probes. The developed genosensor was first optimized using inosine-substituted probes and wild-type, mutant, and non-complementary synthetic targets. Many parameters such as probe concentrations, target concentrations, hybridization buffer, washing buffer, and washing time were optimized to increase the difference in the levels of guanine oxidation signal from wild-type and mutant synthetic targets. Then, the PCR products from the CRISPR-Cas9-edited cells and wild-type cells were tested under optimized conditions. Substantially different levels of guanine oxidation signals were obtained for edited and wild-type PCR products showing that the optimized nanogenosensor was able to detect mutations performed in the *CIZ1* gene with sufficient sensitivity and specificity.

Higher levels of guanine oxidation signals were obtained with PCR amplicons than with 21-base synthetic targets (Figure 5). This is because PCR amplicons contain many guanines outside of the 21-base target site that matches the probe, resulting in an increment of guanine oxidation signals. However, even several guanines in 21-base synthetic targets yielded sufficient levels of signal, and the signals obtained from matched and mismatched synthetic targets could easily be distinguished. When 554-bp PCR products were used, the guanine oxidation signals increased; however, there was still a significant difference in guanine oxidation signals obtained from edited and wild-type PCR products. These data show that the results are similar to 21-base synthetic targets and 554-bp PCR products, suggesting that the working principle of the genosensor is not dependent on the PCR amplicon length.

The genosensor developed here has potential applications in the evaluation of CRISPR-mediated genome editing results. For example, in any HDR-based gene editing assay, the edited clones can be precisely detected by sequencing all the clones; however, this is time-consuming and expensive. Using this genosensor, correctly edited clones can be quickly selected from among tens or hundreds of clones prior to precise verification by sequencing. Moreover, the use of nanogenosensor can be exploited for the detection of single point mutations that cause disease and for detection of CRISPR-Cas9-mediated reversion of the mutation. This method can also be further developed for the detection of other types of CRISPR-based manipulations, including small insertions and deletions and multiple base substitutions generated by HDR, base editing, or prime editing. This approach is also suitable for expansion by employing an array system of sequence-specific probes to detect multiple gene editing outcomes. Further studies could be designed to detect off-target mutations caused by HDR, NHEJ, or other CRISPR-based tools to screen genome editing outcomes more comprehensively.

Although promising, this study includes some limitations. The results are preliminary, and only the *CIZ1* gene was edited using CRISPR-Cas9, and the introduced point mutations were detected using the genosensor as a proof-of-concept. Therefore, future work is needed to fully establish this nanogenosensor as a platform to detect CRISPR-based genome editing outcomes. To address these limitations, several other genes will be targeted, and the nanogenosensor will be used to detect other types of mutations such as multiple base substitutions, insertions, and deletions introduced via CRISPR/Cas9-HDR, base editing, and prime editing. Another limitation of this genosensor is that it may not work efficiently if the GC content of the edited DNA site is quite low. To prevent this, localization of the probe should be optimized such that it matches at least several guanines. Further investigation of this system will provide a basis for exploring other potential applications of the method.

## 5. Conclusions

Here, CRISPR-Cas9 was used to edit the *CIZ1* gene, and the presence of the mutations was confirmed based on electrochemical nanogenosensor by monitoring guanine oxidation signals using disposable pencil graphite electrodes. Some parameters, such as probe concentration, target concentration, hybridization buffer, and washing time, were also optimized in order to improve the performance of the nanogenosensor. The presence of DNA hybridization on the pencil graphite electrodes was confirmed by the observation of an increase in guanine oxidation signal. The irreversible oxidation signal of the guanine base at +1.0 V vs. Ag/AgCl reference electrode was monitored using the DPV technique. The detection method relies on the measurement of increased oxidation currents of the guanine bases only in the presence of the hybrid due to the electrochemical inactivity of the inosine base in the probe sequence. Thus, a simple, sensitive, selective, non-time-consuming and cost-effective electrochemical-based “yes/no” detection system was developed. This hybridization signal was able to differentiate between non-complementary, complementary, and mismatched sequences. The hybridization signal of non-complementary DNA was relatively small because due to minimal interactions. This study has, for the first time, demonstrated direct measurement of CRISPR-Cas9-mediated mutations within a target gene using a nanogenosensor.

## Data Availability

Data available from the corresponding authors P.K. and M.O.

## Supplementary Materials

The following are available online at https://www.mdpi.com/2079-6374/11/1/17/s1, Figure S1: Histograms of optimized conditions in order to maximize the genosensors selectivity. Figure S2: Histogram chart of guanine oxidation current peaks obtained from DPV measurements and comparison with bare PGE surfaces, ssDNA immobilized PGEs and ssDNA wrapped-CNT immobilized PGEs. Table S1: The effect of various optimization conditions (probe concentrations) on the analytical response of FM/MM and FM/NC ratios for the proposed genosensors. Table S2: The effect of various optimization conditions (target concentrations) on the analytical response of FM/MM and FM/NC ratios for the proposed genosensors. Table S3: The effect of various optimization conditions (hybridization buffer) analytical response of FM/MM and FM/NC ratios for the proposed genosensors. Table S4: The effect of various optimization conditions (washing buffer) on the analytical response of FM/MM and FM/NC ratios for the proposed genosensors. Table S5: The effect of various optimization conditions (washing time) on the analytical response of FM/MM and FM/NC ratios for the proposed genosensors. Table S6: Mean and RSD values by means of FM-MM-NC results of both synthetic and PCR amplicons under optimized conditions.
