# Supplementary material for: Detection of CRISPR-Cas9-Mediated Mutations Using a Carbon Nanotube-Modified Electrochemical Genosensor
Source: Biosensors (Basel). 2021 Jan 8;11(1):17. doi: 10.3390/bios11010017 (PMC7827051; doi:10.3390/bios11010017)
Supplement: Supplementary file 1 [file biosensors-11-00017-s001.pdf]

Supplementary Information

Detection of CRISPR–Cas9 Mediated Mutations Using a Carbon Nanotube-Modified Electrochemical Genosensor

Ezgi Kivrak [1], Tekle Pauzaite [2,§], Nikki A. Copeland [2], John G. Hardy [3,4], Pinar Kara [1], Melike Firlak [3,5], Atike I. Yardimci [6], Selahattin Yilmaz [6], Fahreddin Palaz[7] and Mehmet Ozsoz [8]

[1] Department of Analytical Chemistry, Faculty of Pharmacy, Ege University, Izmir, 35100, Turkey.
[2] Department of Biomedical and Life Sciences, Faculty of Health and Medicine, Lancaster University, Lancaster, Lancashire, LA1 4YQ, UK.
[3] Department of Chemistry, Faculty of Science and Technology, Lancaster University, Lancaster, Lancashire, LA1 4YB, UK.
[4] Materials Science Institute, Lancaster University, Lancaster, Lancashire, LA1 4YB, UK.
[5] Department of Chemistry, Gebze Technical University, Gebze, Kocaeli 41400, Turkey.
[6] Department of Chemical Engineering, Izmir Institute of Technology, İzmir, 35430, Turkey.
[7] Faculty of Medicine, Hacettepe University, Ankara, 06100, Turkey.
[8] Near East University, Faculty of Engineering, Lefkosa TRNC, Via Mersin 10, Turkey.
[§] Present address: Cambridge Institute of Therapeutic Immunology & Infectious Disease (CITIID), Jeffrey Cheah Biomedical Centre, Cambridge Biomedical Campus, University of Cambridge, Cambridge CB2 0AW, UK.

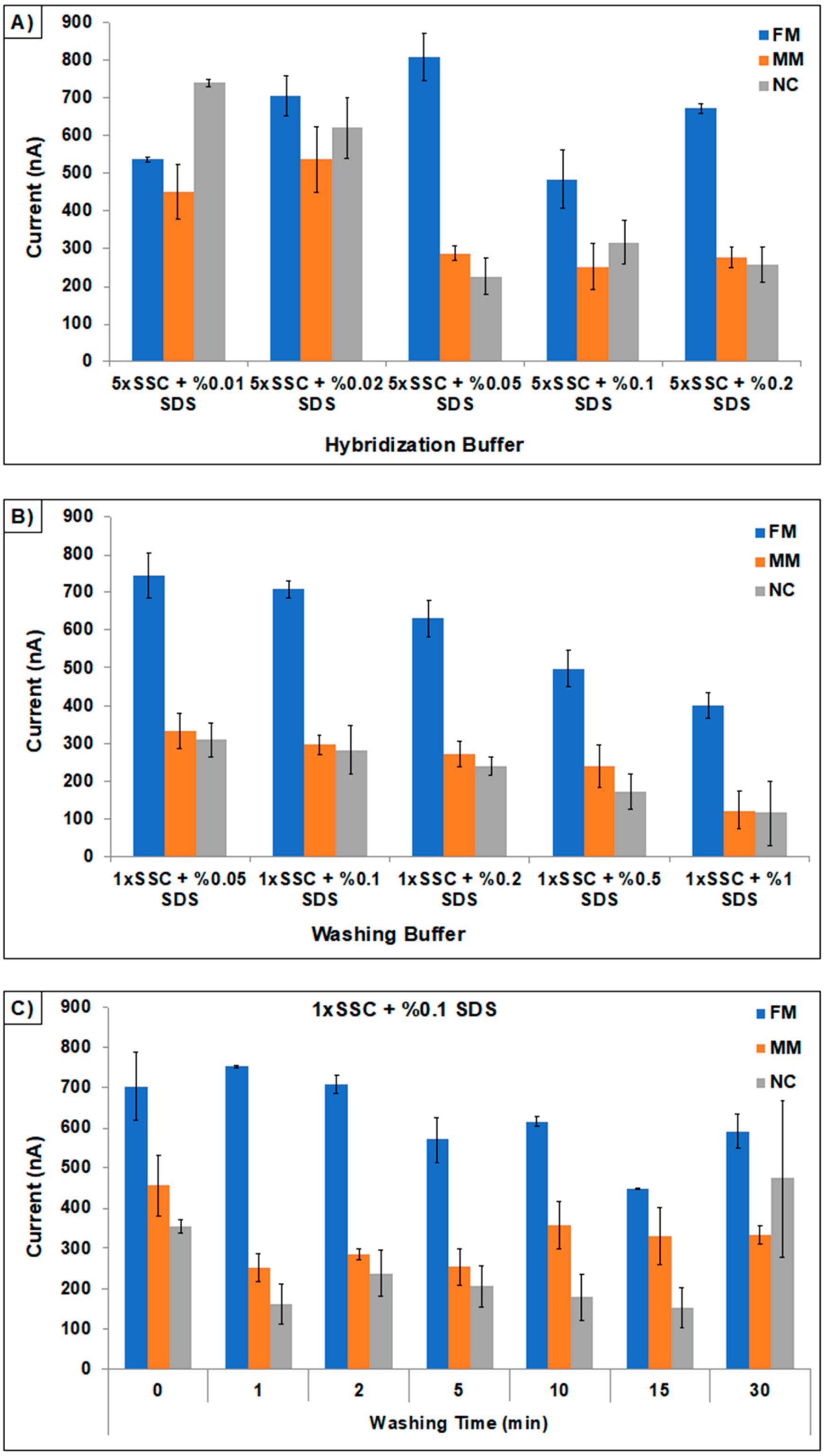

Figure S1. Histograms of optimized conditions in order to maximize the genosensors selectivity. After determining optimum conditions for both probe (5 µg/mL) and target (5 µg/mL) concentrations, the effects of the A) hybridization buffer, B) washing buffer and C) washing time was investigated. For each parameter probe and target concentrations were kept constant at the optimized conditions.

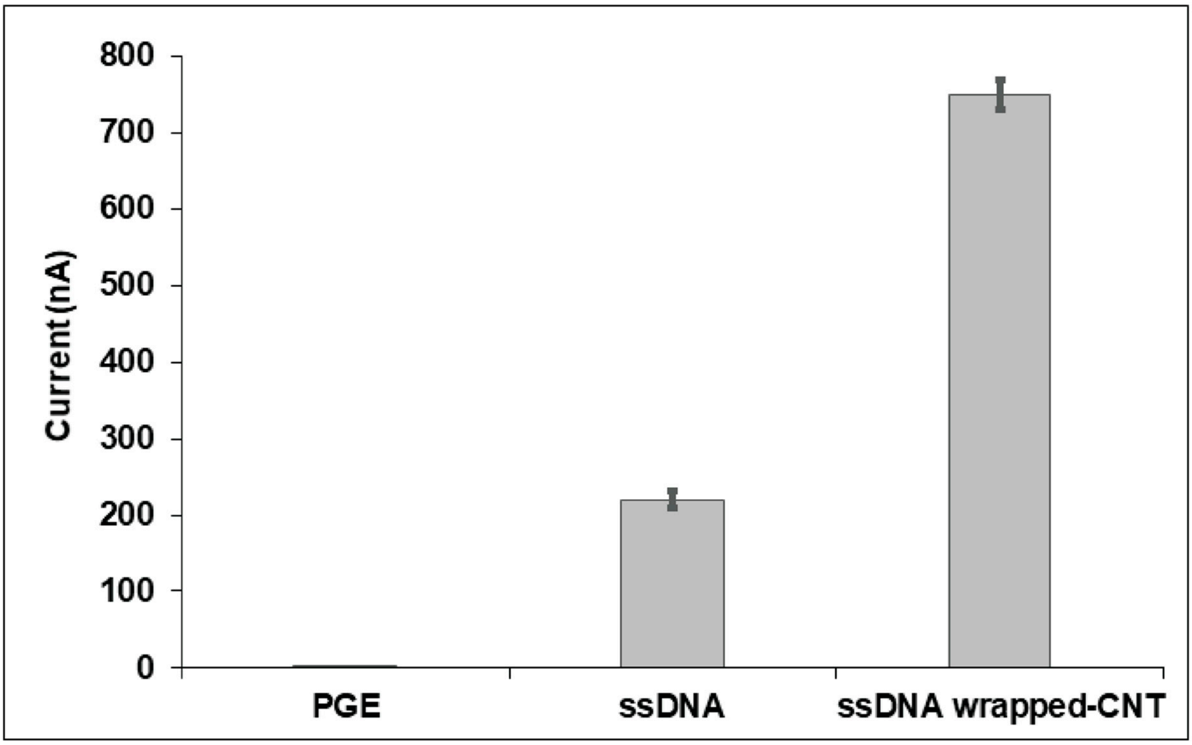

Figure S2. Histogram chart of guanine oxidation current peaks obtained from DPV measurements and comparison with bare PGE surfaces, ssDNA immobilized PGEs and ssDNA wrapped-CNT immobilized PGEs.

Table S1. The effect of various optimization conditions (probe concentrations) on analytical response of FM/MM and FM/NC ratios for the proposed genosensors. Underlined values are attributed to the optimized conditions for each parameter.

	Probe concentrations (μg/mL)						
	0.1	0.2	0.5	1	2	5	10
FM/MM	1.77	1.41	1.54	1.53	1.60	2.15	2.07
FM/NC	1.22	1.28	1.11	1.17	1.22	2.96	1.26

Table S2. The effect of various optimization conditions (target concentrations) on analytical response of FM/MM and FM/NC ratios for the proposed genosensors. Underlined values are attributed to the optimized conditions for each parameter.

	Target concentrations (μg/mL)								
	0.1	0.2	0.5	1	2	5	10	15	20
FM/MM	3.40	2.25	1.48	2.14	1.99	2.33	2.48	2.15	2.16
FM/NC	2.52	1.75	1.77	1.80	2.71	1.91	1.79	1.61	1.94

Table S3. The effect of various optimization conditions (hybridization buffer) analytical response of FM/MM and FM/NC ratios for the proposed genosensors. Underlined values are attributed to the optimized conditions for each parameter.

	Hybridization buffer				
	5xSSC + %0.01 SDS	5xSSC + %0.02 SDS	5xSSC + %0.05 SDS	5xSSC + %0.1 SDS	5xSSC + %0.2 SDS
FM/MM	1.19	1.26	2.72	1.92	1.98
FM/NC	0.84	1.17	3.73	1.53	2.73

Table S4. The effect of various optimization conditions (washing buffer) on analytical response of FM/MM and FM/NC ratios for the proposed genosensors. Underlined values are attributed to the optimized conditions for each parameter.

	Washing buffer				
	1xSSC + %0.05 SDS	1xSSC + %0.1 SDS	1xSSC + %0.2 SDS	1xSSC + %0.5 SDS	1xSSC + %1 SDS
FM/MM	2.23	2.43	2.31	2.07	3.24
FM/NC	2.40	2.77	2.61	2.90	3.45

Table S5. The effect of various optimization conditions (washing time) on analytical response of FM/MM and FM/NC ratios for the proposed genosensors. Underlined values are attributed to the optimized conditions for each parameter.

	Washing time (minutes)					
	0	1	2	5	10	30
FM/MM	1.54	2.98	2.48	2.25	1.72	1.77
FM/NC	1.98	4.66	2.97	2.76	3.46	1.29

Table S6. Mean and RSD values by the means of FM-MM-NC results of both synthetic and PCR amplicons under optimized conditions.

		**Mean (nA)**	**RSD (%)**
**Synthetic sequence**	**FM**	753.00	0.38
	**MM**	252.67	14.05
	**NC**	161.67	30.16
**PCR Amplicon**	**FM**	1385.00	3.03
	**MM**	430.45	11.65
	**NC**	206.33	24.91
